# Portal vein thrombosis associated with COVID-19: points to consider

**DOI:** 10.1259/bjrcr.20200089

**Published:** 2020-07-24

**Authors:** Seyed Hamed Jafari, Razieh Naseri, Neda Khalili, Sara Haseli, Mahbobeh Bahmani

**Affiliations:** 1 Medical Imaging Research Center, Shiraz University of Medical Sciences, Shiraz, Iran; 2 School of Medicine, Tehran University of Medical Sciences, Tehran, Iran; 3 Chronic Respiratory Diseases Research Center, National Research Institute of Tuberculosis and Lung Diseases (NRITLD), Shahid Beheshti University of Medical Sciences, Tehran, Iran

## Abstract

The rapid global spread as well as the mortality and morbidity associated with COVID-19 has raised increasing concern around the globe. Studies have reported that patients infected with the novel coronavirus are prone to coagulopathy. However, information on portal vein thrombosis in patients with COVID-19 is scarce. In this case report, we depict the abdominal CT findings of a 26-year-old male patient with COVID-19 who developed severe abdominal pain during hospitalization and was later diagnosed with portal vein thrombosis. We also demonstrate the chest CT findings of the same patient, which revealed bilateral pleural effusion, a less common imaging finding, and multifocal patchy consolidations. This paper emphasizes that physicians, particularly radiologists, should be aware of thromboembolic events when examining any suspected patient during the current outbreak.

## Introduction

Currently, many countries around the world are facing the coronavirus disease-2019 (COVID-19) pandemic. While the first reports from China showed that manifestations such as fever, dry cough and dyspnoea were the most common presenting features in patients with COVID-19, other unanticipated extra-respiratory symptoms, especially hyper-coagulation conditions affecting different organs, have been increasingly recognized.^1–3^ Herein, we present a patient with COVID-19 who developed portal vein thrombosis during hospitalization. Written informed consent was obtained from the patient for publication of this case report.

## Case presentation

**Figure 1.**
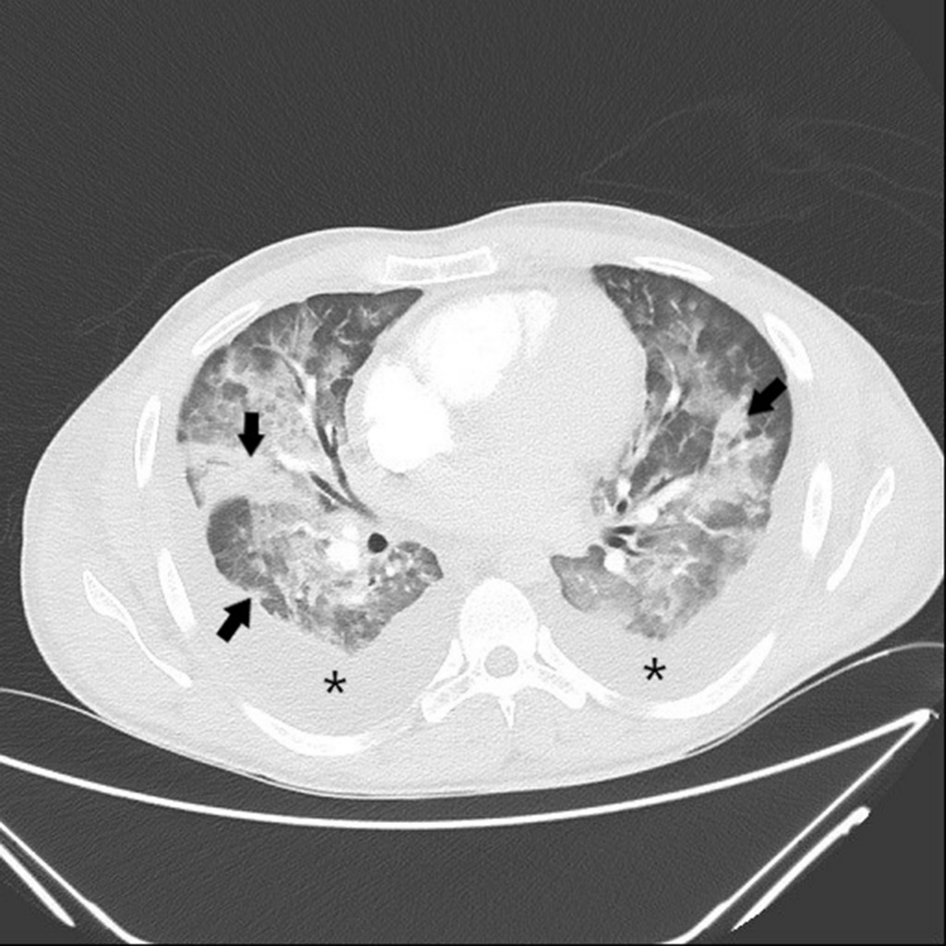
Axial contrast-enhanced CT scan of the chest shows bilateral pleural effusion (*) and multifocal patchy consolidations (black arrows).

**Figure 2.**
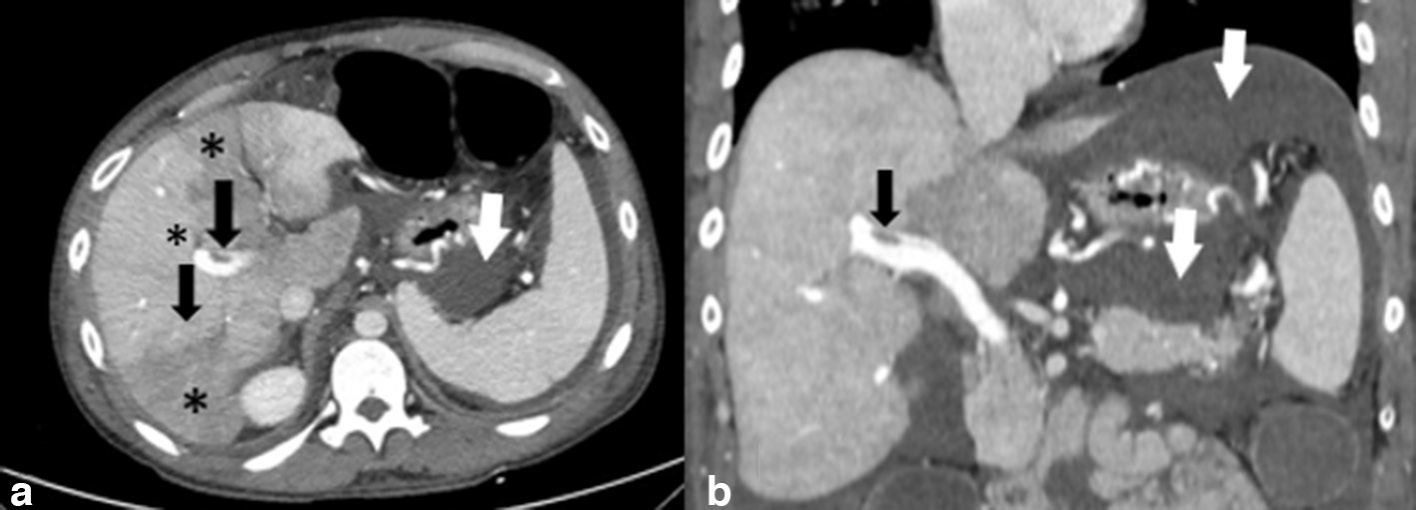
Axial (a) and coronal (c) contrast-enhanced CT images of the abdomen demonstrate a clot within the portal vein (black arrows). There are altered patchy areas of hepatic parenchymal enhancement (*) that are suggestive of occlusion of small branches of the portal vein. Intraperitoneal fluid is also seen on CT images (white arrows).

A previously healthy 26-year-old male was admitted to our hospital complaining of a 2-day history of respiratory distress and fatigue. The patient denied any recent travel and had no positive contact history during the last months. His past medical history was unremarkable except for a history of controlled asthma diagnosed 4 years ago. He did not take any medications and had no history of tobacco smoking or alcohol consumption. On admission, the patient was haemodynamically stable with a blood pressure of 115/75 mmHg and a pulse rate of 88 bpm. He had no fever (oral temperature = 37.1°C), but had an increased respiratory rate of 33 breaths/min and an oxygen saturation of 92% on room air. Blood tests revealed normal leukocyte and lymphocyte count (7.2×10^3^/µl and 2800/µl, respectively), elevated C-reactive protein (CRP) (96 mg l^−1^), prolonged prothrombin time (PT) (39 s) and international normalized ratio (INR) (1.34) and increased D-dimer level (0.5 mcg/ml). The patient tested positive for SARS-CoV-2 by reverse transcription polymerase chain reaction (RT-PCR) and had findings suggestive of COVID-19 on chest CT scan, including multifocal patchy consolidations and bilateral pleural effusion (Figure 1). He was later admitted to the intensive care unit (ICU) due to severe respiratory distress. While receiving care in the ICU, he developed severe abdominal pain located in the right upper quadrant on the fifth day of admission and thus underwent contrast-enhanced abdominal CT scan for further evaluation. Multiphasic CT scan (including an arterial phase, a portal venous phase and an equilibrium phase) on a 16-slice multidetector CT scanner was performed for the patient after injection of 80–120 cc of iodinated contrast media. Portal vein thrombosis was detected in the portal venous phase of abdominal CT. In addition, intraperitoneal fluid was seen on CT images, which was likely due to portal hypertension caused by portal vein thrombosis (Figure 2). Immediate anticoagulation therapy with continuous intravenous heparin infusion (1000 U/h) was initiated and with the improvement of his medical condition, the patient was discharged after 9 days of hospitalization.

## Discussion

Patients with COVID-19 experience a hypercoagulable state, either in the arterial or the venous system, as demonstrated by thrombocytopenia, elevated D-dimer levels and prolonged PT.^4^ There are several possible explanations for the greater risk of coagulopathy in patients with COVID-19, including endothelial dysfunction, von-willebrand factor elevation, toll-like receptor and tissue factor pathway activation, as a result of systemic inflammation, platelet-leukocyte aggregation, and also activation of macrophages, monocytes, endothelial cells, platelets and lymphocytes in response to a viral infection.^4^ Hypoxia, immobilization and disseminated intravascular coagulation (DIC) have also been proposed as the potential causes of hypercoagulability in COVID-19 infection.^5^

Many studies have reported thromboembolic complications associated with COVID-19 infection.^6–9^ In a recent study by Klok et al, the incidence of thrombotic complications in critically ill patients with COVID-19 was reported to be as high as 31% and pulmonary embolism (PE) was the most common thrombotic complication among all patients.^5^ However, only one case report is present in the literature that has described a patient with suspected COVID-19, but no RT-PCR confirmation, who developed portal vein thrombosis during hospitalization.^10^

We report a young male patient with COVID-19 who was diagnosed with portal vein thrombosis after developing acute abdominal pain. Considering the lack of predisposing risk factors for venous thromboembolism in the case described here, we believe that the known hypercoagulability-vasculitis state in COVID-19 may play a role in this patient. Similarly, other reports have described patients with COVID-19 who were found to have PE without any risk factors.^11,12^ Also, male gender and invasive mechanical ventilation may be associated with thromboembolic events, as mentioned by Grillet and colleagues.^13^

## Learning points

- Patients with confirmed COVID-19 are at greater risk of thromboembolic events.
- The coagulation profile of hospitalized patients should be carefully monitored.
- The use of prophylactic anticoagulants should be considered in hospitalized patients with COVID-19, unless medically contraindicated.
- We strongly recommend abdominal imaging, especially contrast-enhanced studies, for critically ill patients suffering from severe abdominal pain, either as the initial presenting symptom or during hospitalization.
